# Antibiotic Biosynthesis Pathways from Endophytic *Streptomyces* SUK 48 through Metabolomics and Genomics Approaches

**DOI:** 10.3390/antibiotics10080969

**Published:** 2021-08-12

**Authors:** Mohd Shukri Baba, Noraziah Mohamad Zin, Siti Junaidah Ahmad, Noor Wini Mazlan, Syarul Nataqain Baharum, Nuraziemah Ahmad, Fazren Azmi

**Affiliations:** 1 Department of Biomedical Science, Kulliyyah of Allied Health Sciences, International Islamic University, Kuantan 25200, Malaysia; mohd_shukri@iium.edu.my; 2 Center of Diagnostics, Therapeutics & Investigations, Faculty of Health Sciences, Universiti Kebangsaan Malaysia, Jalan Raja Muda Abdul Aziz, Kuala Lumpur 50300, Malaysia; 3 Faculty of Health Sciences, University of Sultan Zainal Abidin, Kuala Nerus 21300, Malaysia; junaidahahmad@unisza.edu.my; 4 Analytical and Environmental Chemistry, Faculty of Science and Marine Environment, Universiti Malaysia Terengganu, Kuala Nerus 21030, Malaysia; noorwini@umt.edu.my; 5 Institute of Systems Biology, Universiti Kebangsaan Malaysia, Bangi 43600, Malaysia; nataqain@ukm.edu.my; 6 Center of Drug Delivery Technology, Faculty of Pharmacy, Universiti Kebangsaan Malaysia, Jalan Raja Muda Abdul Aziz, Kuala Lumpur 50300, Malaysia; p94276@siswa.ukm.edu.my (N.A.); fazren.azmi@ukm.edu.my (F.A.)

**Keywords:** *Streptomyces* sp. SUK 48, biosynthesis pathways, non-toxic isolates, bioinformatics, metabolites profiles

## Abstract

*Streptomyces* sp. has been known to be a major antibiotic producer since the 1940s. As the number of cases related to resistance pathogens infection increases yearly, discovering the biosynthesis pathways of antibiotic has become important. In this study, we present the streamline of a project report summary; the genome data and metabolome data of newly isolated *Streptomyces* SUK 48 strain are also analyzed. The antibacterial activity of its crude extract is also determined. To obtain genome data, the genomic DNA of SUK 48 was extracted using a commercial kit (Promega) and sent for sequencing (Pac Biosciences technology platform, Menlo Park, CA, USA). The raw data were assembled and polished using Hierarchical Genome Assembly Process 4.0 (HGAP 4.0). The assembled data were structurally predicted using tRNAscan-SE and rnammer. Then, the data were analyzed using Kyoto Encyclopedia of Genes and Genomes (KEGG) database and antiSMASH analysis. Meanwhile, the metabolite profile of SUK 48 was determined using liquid chromatography-mass spectrophotometry (LC-MS) for both negative and positive modes. The results showed that the presence of kanamycin and gentamicin, as well as the other 11 antibiotics. Nevertheless, the biosynthesis pathways of aurantioclavine were also found. The cytotoxicity activity showed IC50 value was at 0.35 ± 1.35 mg/mL on the cell viability of HEK 293. In conclusion, *Streptomyces* sp. SUK 48 has proven to be a non-toxic antibiotic producer such as auranticlavine and gentamicin.

## 1. Introduction

*Streptomyces* sp. was known as an antibiotic producer 80 years back [1]. *Streptomyces* was the large group of genus Actinomycetes and filamentous aerobic Gram-positive bacteria [2,3] and was known to produce many remarkable secondary metabolites that can have antibacterial, antifungal, antiplasmodial, and antiviral functions [4,5]. The distinctiveness of the *Streptomyces* sp. genome is the fact that its biosynthetic genes cluster encodes enzymes that responsible for secondary metabolites production [6]. For example, *Streptomyces kebangsaanensis* contains *phz* enzymes to produce novel metabolite, 6-((2-hydroxyl-4-metoxylphenoxylcarbonyl)phenazine-1-carboxylic acid HCPCA) (tubermycin B) which acts as an antibacterial agent [7,8]. Coronamycin was reported by Strobel et al. in 2004, isolated from endophytic *Streptomyces* sp. from Monstera sp. in Amazon, Peru which acts as an antiplasmodial agent against *Plasmodium falciparum* with the IC_50_ value of 9 ng/mL [4]. In addition, the coronamycin had cytotoxicity effects against primary mammary epithelial cells (HMEC), which was a similar effect to *taxol*, an anti-cancer drug [4].

*Acinetobacter baumanii* is a Gram-negative coccobacilli that causes nosocomial infections such as pneumonia and infections in the urinary tract [9,10]. Frequently being documented as an opportunistic pathogen in humans, *A. baumannii* can potentially affect people with compromised immune systems. Up until now, the natural habitats of *A. baumannii* are still unknown, although this bacteria is almost exclusively isolated from the hospital environment and occasionally it has been found in environmental soil and water samples [11,12]. *A. baumannii* is one of the members of ESKAPE pathogen group that consists of *Enterococcus faecium, Staphylococcus aureus, Klebsiella pneumoniae, Acinetobacter baumannii, Pseudomonas aeruginosa,* and *Enterobacter* spp. ESKAPE was identified as a group of pathogens with the high rate of antibiotic resistance that is responsible for the increased cases of nosocomial infections [13]. There are so many documents that reported that the multidrug resistance (MDR) of *A. baumannii* has spread to the hospitals and many health settings due to the usage of medical facilities and instruments. It also has been reported multiple times that in the current COVID-19 pandemic, secondary infection of *A. baumannii* and other microbes could occur in SARS-CoV-2-infected patients [14].

The intense efforts focusing on the search for new antibiotics and antimicrobial agents based on natural products have gained significance among researchers around the world. This scenario is even more prominent when the modern medical world is now faced with the conflict of increasing bacterial resistance to the current use of antibiotics. As part of this journey, several methods have been proposed to investigate the antibacterial activities, elucidate their modes of action, identify their targets in the bacterial cell, and investigate their future therapeutic uses as novel drugs [15]. As a result, few new antibiotic classes have been discovered in the last several decades [16]. Recent developments in omics technologies such as metabolomics, transcriptomics, genomics and proteomics have been widely documented where they are often associated with innovative breakthroughs in bioinformatics, genomic sequencing and analytical instrumentation such as mass spectrometry and LC-MS. Omics technology has also provided important approaches to processes related to bacterial virulence and physiology as well as mechanisms of antimicrobial agents [17,18].

Metabolomics is one of the omics studies, defined as the study of the global profile of metabolites present in a biological system under certain conditions and time [19]. Metabolites is a term referring to a range of end products of cellular processes that belong to different classes such as amino acids, sugars, organic acids, steroids, nucleic acid bases, fatty acids, sugar alcohols and many more [20]. Beginning in the 2000s, metabolomics approaches have been widely applied to determine and investigate the response of microorganisms to various external stresses and environmental factors such as extreme temperature and pH and the action of organic compounds [21]. In addition, many previous studies have evidenced that a metabolomics approach is an appropriate tool for the study of bacteria metabolic disorders treated with antibiotics and many types of bioactive compounds [22]. However, metabolomics approaches for the exploration and discovery of mechanisms of action by bioactive compounds derived from plants and endophytic microorganisms have yet to be fully explored. By using *Staphylococcus aureus* as the most commonly studied bacteria for this purpose, in essence, the resulting metabolic profile of the tested bioactive compounds can often be compared to profiles obtained using antibiotics, whereby the mechanisms of action are well known [22].

For instance, in 2007, Yu and his colleagues documented that anti-*S. aureus* action of *Aquilegia oxysepala* extract and its major chemical components such as maguoflorine, berberine, genkwanin and apigenin were investigated using metabolomics approaches (DAD/HPLC/ESI-MS analysis combined with PCA). This method is compared with the induced profile of nine antibiotics which the modes of action were known. This study has shown that *A. oxysepala* targets are similar to several protein synthesis inhibitors such as erythromycin, streptomycin and chloromycetin. This study also demonstrates that barberine had similar effects to two nucleic acid target drugs; rifampicin and norfloxacin [22]. In another study, jatrorrhizine and palmatite were found as the most active compounds when the intracellular metabolic profile of *S. aureus* was treated with rhizome extract from *Tinospora capillipes* and its major chemical components such as tinoside, jatrorrhizine, columbin and palmatine were investigated with HPLC-ESI/MS application. In this study, it was evidenced that the inhibition of topoisomerase, gyrase and RNA polymerase was identified as the mechanisms of action [23]. Previously, a study applying metabolomics profiling revealed that triphenylbismuthdichloride successfully influenced the catabolism of bacterial pyruvate which in turn led to the discovery of this compound as a highly effective and uncompetitive inhibitor of bacterial pyruvate dehydrogenase complex. This study also revealed that metabolomics may represent a high-potential strategy to give a clearer picture of mechanisms of action of bioactive compounds and to extend combating multidrug-resistant bacteria [24].

With the availability of all the convenience offered by omics technology, this approach provides a very significant opportunity to determine the antimicrobial mechanisms by the targeted bioactive compound. Additionally, due to the level of structural diversity found in the phytochemistry of the compounds, this approach is highly relevant for the determination of the most outstanding compounds as the therapeutic agents, and at the same time leads to the identification of new targets. Basically, the design of new antibiotics with different modes of mechanisms and structures will also be driven by these studies, thus the occurrence of cross-resistance can be avoided and the development of resistance to these newly found compounds will be delayed. The design of new antibiotics with different action modes and structures will be also driven by these studies, and thus cross-resistance events will be avoided and the development of resistance to these novel compounds will be delayed [20]. Considering all of these factors and examples, it is clearly evidenced that nowadays, omics technology is the most preferred approach in answering the burden of unstoppable problems of bacteria with multidrug-resistant characteristics, considering all the benefits and multifactorial advantages of omics technology given above.

Therefore, the presented study summarizes our project report with the major aim to determine antibiotic biosynthesis pathways of newly isolated Streptomyces sp. SUK 48 as well as the antibacterial activity and toxicity test of crude extracts of the isolate.

## 2. Materials and Methods

### 2.1. Brasilia sp. Collection

Endophytic *Streptomyces* sp. SUK 48 was isolated from the fruit of *Brasilia* sp. located at 2.9125 N 101.787 E at Universiti Kebangsaan Malaysia reserve forest.

### 2.2. Chemicals

All chemicals and reagents used in this study were of high analytic grade, purchased from Sigma-Aldrich (Kuala Lumpur, Malaysia) and Merck (Kuala Lumpur, Malaysia) such as ethyl acetate, PBS, Phosphate-buffered saline (pH 7.2), methanol 99.9% (Merck, KL, USA), acetonitrile (Merck, KL, USA), DMSO (Dimethyl sulfoxide).

### 2.3. Genomic DNA Extraction and Whole Genome Sequencing

*Streptomyces* sp. SUK 48 was obtained from the fruit of *Brasilia* sp. located from Bangi Reserved Forest of UKM [25]. Genomic DNA extraction was performed as previously described (Promega, Madison, WI, USA). The purity of DNA was determined using Nanodrop 2000 machine (Thermo Fisher Scientific, Waltham, MA, USA). The presence of actinomycete DNA was also detected using 0.6% agarose gel and examined under UV light (Aplegen Omega Fluor, San Francisco, CA, USA). The genome sequencing was performed by using the SMRT RS II Pacific Bioscience Technology platform (Treecode Inc., Singapore).

### 2.4. Bioinformatic Analysis

Genomic raw data were processed as described, with several modifications [26]. Sequencing data were pre-processed, de novo assembled and polished using SMRT Link v6.0.0 command line pbsmrtpipe. The whole genome was then assembled using the Hierarchical Genome Assembly Process 4.0 (HGAP 4.0) [27,28]. The polished genome was used as an input for structural annotation. The tRNA was predicted to use tRNAscan-SE v1.3.1 [29]. The rRNA prediction was then formed using rnammer v1.2. [28,30]. Antibiotics and Secondary Metabolite Analysis Shell (AntiSMASH Version 5, Selangor, Malaysia, https://antismash.secondarymetabolites.org) was used to identify genes that encode antibiotics and secondary metabolites from the raw data by comparing with the genes reference within the database through online platform http://antismash.secondarymetabolite.org [31,32]. The genomics and metabolomics analysis was determined by using the Kyoto Encyclopedia of Genes and Genomes (KEGG) (http://www.genome.jp/kegg/).

For detection of a specific protein in the aurantioclavine production, several specific amino acid sequences have been selected to be blasted with the protein from Penicillium expansum (taxid: 27334) by using PSI-BLAST (position-specific iterative-basic local alignment search tool) algorithm [33]. The sequence of amino acid ID quiver 618 for methyltransferase enzyme and amino acid ID quiver 4096 for aurantioclavine synthase enzyme is used.

### 2.5. Liquid Chromatography-Mass Spectrometry (LC-MS) Analysis

About 3 mg/mL extracts of S48D14 (SUK 48 day fermentation time day 14th), were sent for LC-MS analysis [34]. Chromatographic separation was performed on the Thermo Scientific column C18 (AcclaimTM Polar Advantage II, Waltham, MA, USA, 3 × 150 mm, 3 μm particle size) on the UltiMate 3000 UHPLC system (Dionex, Sunnyvale, CA, USA). The elution rate for the gradient was 0.4 mL/min at 40 °C using (A) 0.1% formic acid water and (B) 100% acetonitrile, with a total runtime of 22 min. The gradient started at 5% solvent B for 3 min (0–3 min), then the gradient increased to 80% solvent B for 7 min (3–10 min) and remained at 80% solvent B for 5 min (10–15 min). At the end of the day, the gradient returned to 5% solvent B in 7 min (15–22 min). High-resolution MS was performed using MicrOTOF-Q III (Bruker Daltonic, Bremen, Germany) using ESI positive and negative ionization with the following settings: capillary voltage at 4500 V; nebulizer pressure at 1.2 bar; and drying gas flow at 8 L/min at source temperature at 200 °C; and *m*/*z* at 50 to 1000 Da.

### 2.6. Mass Spectrometry (MS) Data Handling

The MS raw data were obtained in “d format” from Bruker Compass DataAnalysisViewer version 4.2 (Bruker Daltonics, Bremen, Germany) before being imported into Profile Analysis 2.0 software (Bruker Daltonic, Bremen, Germany) for data bucketing [35,36,37]. “Find Molecular Features” was applied to the raw data to assist in molecular compound identification, which was carried out using the Find Molecular Features (FMF) approach. Signal/noise threshold: 5; correlation coefficient: 0.7; minimum compound length: 8; and smoothing width: 2. The compound bucket table was calculated using the advanced bucket function using time alignment parameters. The time range was set to 0.00 to 22.04 min and the mass range was 49 to 1001 *m*/*z*.

### 2.7. Metabolite Identification

The identification of secondary metabolites in the LC–MS analysis was achieved by a mass-based search followed by manual verification. The *m*/*z* value of the molecular ion of interest was searched against online databases, namely METLIN and the Natural Products Dictionary (DNP) [38,39]. Metabolites with molecular weights within the specified tolerance range for the query *m*/*z* value have been retrieved from databases as a putative identification.

### 2.8. In-Vitro Cytotoxicity Test

Cell toxicity tests of human epithelial kidney cells, HEK 293 (Addexbio, San Diego, CA, USA) were conducted as previously described [40]. Briefly, 100 µL of HEK 293 cells with a concentration of 5.5 × 10^4^ cells/mL were seeded into a 96-well plate and incubated at 37 °C with 5% CO2 for 24 h for cell attachment. After 24 h, the spent medium was removed and 100 µL of fresh medium containing SUK 48 extract was pipetted into the wells to give a final concentration at 10-fold dilution starting from 1 mg/mL with 0.1% DMSO. Cells in cell culture media without SUK 48 extract served as the negative control. The plates were further incubated at 37 °C with 5% CO2 for another 48 h. After the incubation, 20 µL of 5 mg/mL MTT dissolved in sterile PBS was added into each well and incubated for 2 h at 37 °C with 5% CO_2_. After another 2 h, the medium was removed, and the formazan crystal formed was resuspended in 100 µL DMSO. DMSO alone was used as blank. Absorbance was read at 570 nm using a microplate reader (NanoQuant Infinite M200 PRO, Tecan, Switzerland). The formula for the percentage of cell viability is shown below:

(1) $$\mathrm{Percentage}\;\mathrm{viability},\%=\;\left(\frac{Absorbance\;extract\;-Absorbance\;blank}{Absorbance\;negative\;-\;Absorbance\;blank}\right)\;\times \;100$$

The concentration of the crude extract that reduced half of the cells’ viability (IC_50_) was measured by executing nonlinear regression along with sigmodial concentration-response curve construction using GraphPad Prism 9 (San Diego, CA, USA).

## 3. Results

**Figure 1:**
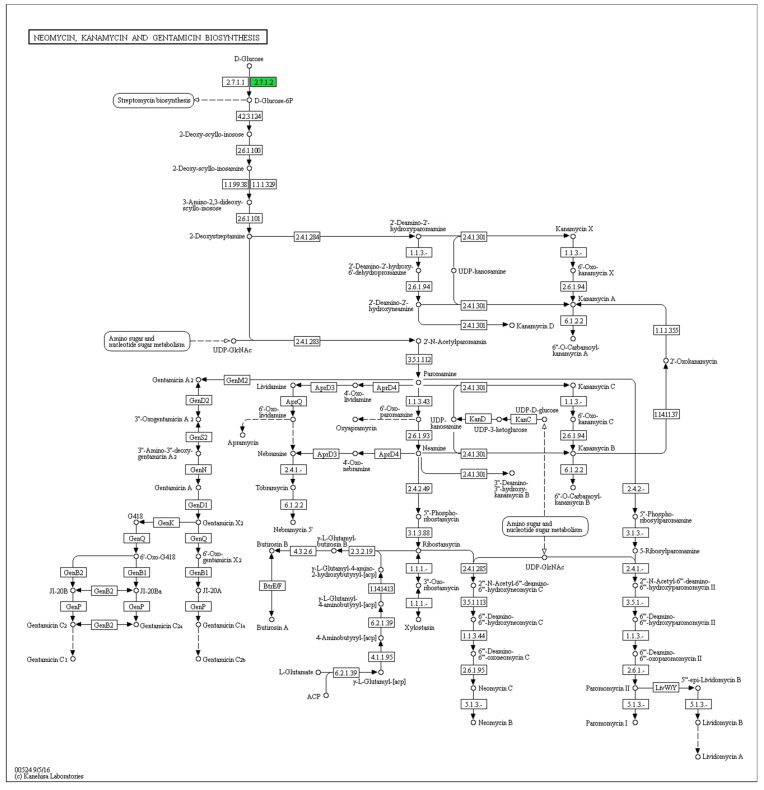
Antibiotic synthesis of neomycin, kanamycin and gentamicin. Source: KEGG database (*Kyoto Encylopedia Genes and Genomes*).

**Figure 2:**
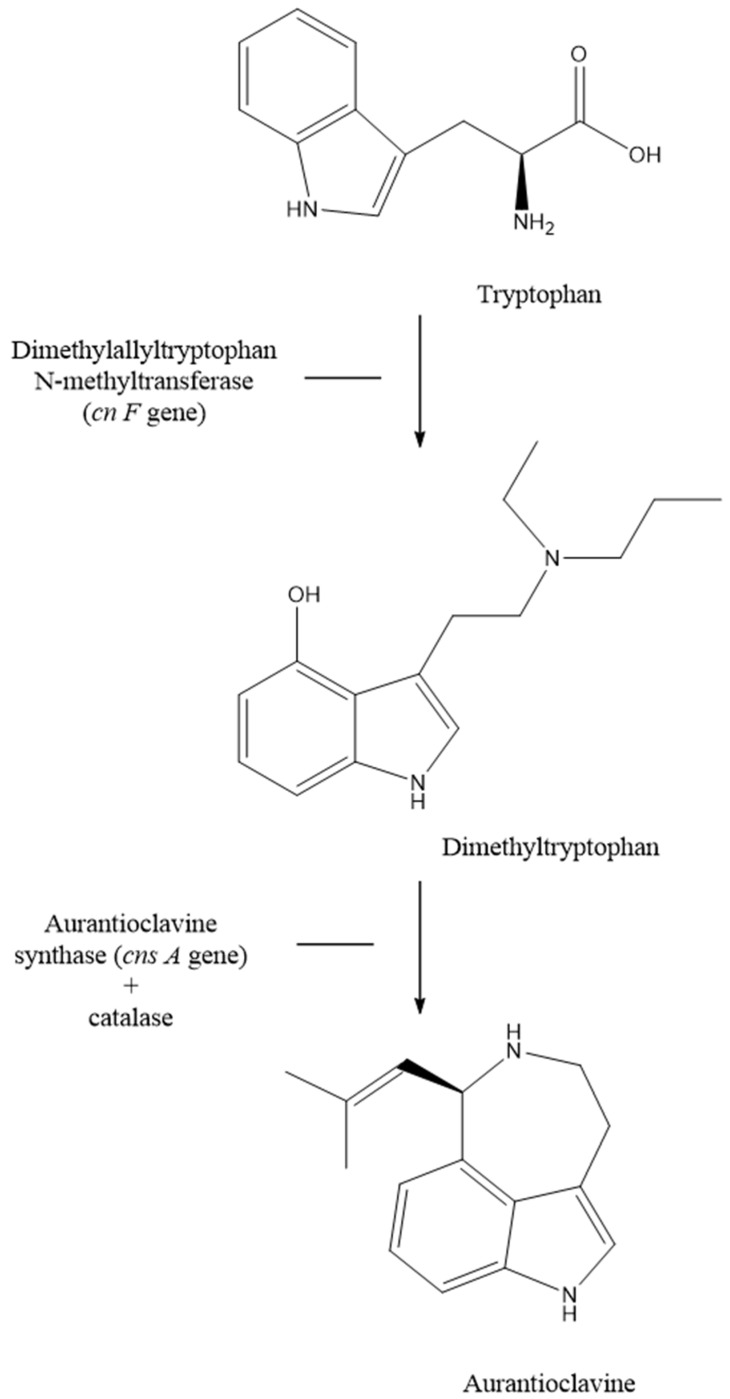
Summary of biosynthesis pathway of aurantioclavine found in *Streptomyces* sp. SUK 48, which was similar with biosynthesis pathway in *Penicillium expansum*.

**Figure 3:**
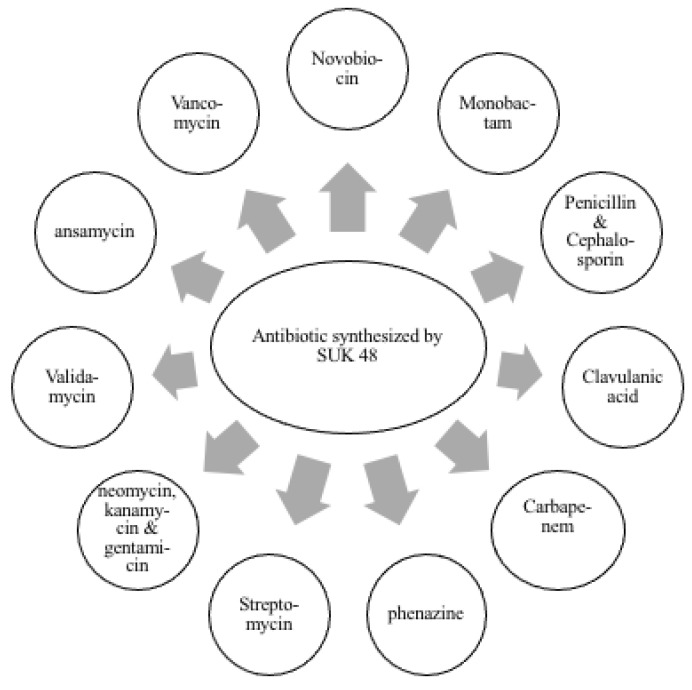
List of antibiotic biosynthesis pathways by *Streptomyces* SUK 48.

The bioinformatics analysis using Kyoto Encyclopedia of Genes and Genomes (KEGG) database (Figure 1) and antiSMASH analysis shows that *Streptomyces* sp. SUK 48 had a pathway of putative kanamycin biosynthesis [41]. This result was supported by position region number one of this biosynthesis in the gene cluster [41]. In addition, instead of in positive mode (Table S1), putative kanamycin A compound (483.2323 *m*/*z*, t = 6.71) and putative gentamicin (492.3036 *m*/*z*, t = 12.26) were found in negative mode (Table S2). On the other hand, a putative aurantioclavine was found in positive mode through metabolomics analysis which was reported as a potential anti-plasmodial agent [42]. Biosynthesis pathways of putative kanamycin and putative gentamicin were illustrated in Figure 1, whereas putative aurantioclavine biosynthesis was illustrated as in Figure 2. As reported, SUK 48 had 11 biosynthesis pathways related to antibiotics which act as antimicrobials, as mentioned in Figure 3.

Additionally, SUK 48 was able to inhibit the growth of *Pseudomonas aeruginosa, Acinobacter baumanii* and *Escherichia coli* at 100% inhibition rate, while 55% inhibits *Bacillus cereus* and 40% inhibit Methicillin-resistant strain of *Staphylococcus aureus* [25].

**Figure 4:**
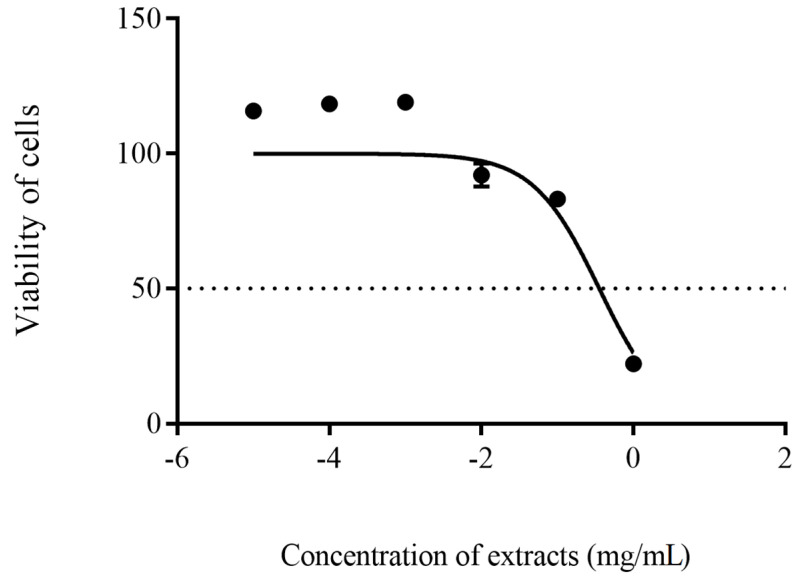
The cytotoxicity activity showed IC_50_ values were at 0.35 ± 1.35 mg/mL on the cell viability of HEK 293. The experiment was carried out in triplicate.

On the other hand, the cell viability of HEK 293 was observed to be concentration- dependent. Crude extract of SUK 48 at a concentration of 1 × 10^−5^–1 × 10^−1^ mg/mL did not cause any toxicity reaction against HEK 293 with cell availability more than 80% after 48 h incubation. Furthermore, its IC_50_ value was at 0.35 ± 1.35 mg/mL, as shown in Figure 4.

## 4. Discussion

Antibacterial activity of SUK 48 had proven its ability to inhibit the growth of *P. aeruginosa, A. baumanii* and *E. coli* by 100%. Since *A. baumanii* strain was collected from the clinical setting and the resistance strain, we hypothesize that SUK 48 may produce other antibiotics or any of their combination that may have inhibited the *A. baumanii* growth.

There were 11 specific biosynthesis pathways of antibiotic production that have been identified in this study (Figure 1). Most of the antibiotics such as penicillin were inhibiting the synthesis of cell wall [26,27,28,29], while novobiocin, phenazine and ansamycin were inhibiting the synthesis of DNA [30,31,32].

As was already documented, the modes of action of streptomycin, neomycin, kanamycin and gentamycin were to inhibit the synthesis of protein in targeted pathogens [33] and as for validamycin, the mode of action was more on inhibition of tyrosinase production [34].

There were 34 gene clusters that were responsible for producing secondary metabolites in *Streptomyces* sp. SUK 48, including polyketide synthase (PKS) type I, II and III, as well as nonribosomal peptide synthase (NRPS) and hybrid NRPS/PKS [41]. PKS genes were encoding polyketide metabolites that had multiple antimicrobials targets, such as tetracycline and antifungal amphotericin B [43,44]. Specifically, PKS type I (PKSI) genes encode the production of macrocyclic polyketides (macrolides) such as avermectin [45,46]. Additionally, PKS type II (PKSII) genes encode the production of aromatic polyketides such as actinorhodin [45,47]. On top of that, PKS type III (PKSIII) genes, which were very rare in bacteria, encoded non-protein-based amino acids such as kendomycin and glycopeptide based antibiotics such as vancomycin [45,48].

In this study, we found that kanamycin and gentamycin were identified in metabolomics analysis and BGC of kanamycin was found in genomics analysis (Figure 1). Kanamycin was first isolated from *Streptomyces kanamyceticus* [48]. Only enzyme glucokinase was detected during the analysis that initiated the reaction of kanamycin and gentamicin biosynthesis. To prove the biosynthesis, the kanamycin gene cluster was found at 1% homolog with the reference cluster indicating the unique cluster of this kanamycin cluster [41]. However, further study on validation of metabolomics data could be carried out by using LC-MS/MS and Nuclear Magnetic Resonance (NMR) to confirm the presence of the metabolites. Additionally, BCG for the kirromycin gene has been identified as two different clusters, which were the 12th cluster with 16% homolog, and the 34th cluster with 3% homolog [41]. Interestingly, these clusters encoded the production of an antiplasmodial compound that inhibits the synthesis of protein in the *Plasmodium falciparum* [42,49]. For the record, the kirromycin had been isolated from *Streptomyces collinus* Tü 365 [49].

The biosynthesis pathways of aurantioclavine (Figure 3) begins with the conversion of tryptophan into dimethyltrptophan with the presence of dimethylallyltryptophan N-methyltransferase that encodes by *cns*F gene. Then, dimethyltryptophan was converts into aurantioclavine with the presence of auranthioclavine synthase that encodes by *cns*A gene and also catalase [50,51]. The production of aurantioclavine was similar with aurantioclavine in *Penicillium expansum* found in nature [50]. Moreover, aurantioclavine was listed as a potential antiplasmodial agent in a recent study [42]. To somehow summarize our presented findings, what makes this project different is the discovery of three antibiotics—kanamycin, gentamicin and auranticlavine—from one single source of endophytic bacteria, namely *Streptomyces* SUK 48, which has unique antimicrobial properties.

The in vitro cytotoxic effect of the SUK 48 crude extract on normal mammalian cells was carried out to assess its potential cytotoxic activity to evaluate their suitability for potential application [52]. Most of the tested concentrations (1 × 10^−5^–1 × 10^−1^ mg/mL) did not show any cytotoxic activity against the tested cells, in which cells viable beyond 80% were considered to have negligible cytotoxicity [53].

## 5. Conclusions

*Streptomyces* sp. SUK 48 was demonstrated to be a potential antibiotic producer capable of inhibiting the growth of bacteria. Through the approaches of metabolomics and genomics, we were able to explore the uniqueness of the biosynthesis of gene clusters and secondary metabolites biosynthesis pathway. More importantly, the biosynthesis of kanamycin, gentamicin and auranticlavine could be discovered from *Streptomyces* SUK 48.

## Data Availability

The genome data in this paper is the under bioproject; PRJNA587018 and available at http://www.ncbi.nlm.nih.gov/bioproject/587018.

## Supplementary Materials

The following are available online at https://www.mdpi.com/article/10.3390/antibiotics10080969/s1, Table S1: Metabolite profiling of *Streptomyces* SUK 48 in positive mode, Table S2: Metabolite profiling of *Streptomyces* SUK 48 in negative mode.
